# PD-L1 Test-Based Strategy With Nivolumab as the Second-Line Treatment in Advanced NSCLC： A Cost-Effectiveness Analysis in China

**DOI:** 10.3389/fonc.2021.745493

**Published:** 2021-12-13

**Authors:** Qiao Liu, Xia Luo, Zhen Zhou, Liubao Peng, Lidan Yi, Xiaomin Wan, Chongqing Tan, Xiaohui Zeng

**Affiliations:** ^1^ Department of Pharmacy, The Second Xiangya Hospital of Central South University, Changsha, China; ^2^ Menzies Institute for Medical Research, University of Tasmania, Hobart, TAS, Australia; ^3^ Department of Nuclear Medicine/PET Image Center, The Second Xiangya Hospital of Central South University, Changsha, China

**Keywords:** cost-effectiveness, NSCLC, nivolumab, PD-L1 test, China

## Abstract

**Objective:**

Our previous economic assessment found that nivolumab was not cost-effective for Chinese patients with advanced non-small cell lung cancer (NSCLC) and without *EGFR* mutations or *ALK* translocations, when compared with the standard second-line drug docetaxel. However, a greater survival benefit with nivolumab was observed for patients with 1% or greater tumor programmed death ligand 1 (PD-L1) expression. In view of this, we designed the present analysis to explore whether it is cost-effective to use the PD-L1 test to guide second-line nivolumab treatment in China.

**Material and Methods:**

A Markov model was established to project the lifetime costs and quality-adjusted life-years (QALYs) of three second-line treatment strategies: nivolumab and docetaxel (strategies without a PD-L1 test) and PD-L1 test-based strategy. Deterministic and probabilistic sensitivity analyses were performed to examine the robustness of our results. Additional price reduction and willingness-to-pay (WTP) threshold scenario analyses were performed to explore the impact of economic and health policies with Chinese characteristics on our results.

**Results:**

The PD-L1 test-based strategy costs approximately CNY 194,607 (USD 28,210) or more and yielded an additional 0.27 QALYs compared to the docetaxel strategy without a PD-L1 test, equating an incremental cost-effectiveness ratio (ICER) of CNY 731,089 (USD 105,978)/QALY. Deterministic sensitivity analyses showed that the price of nivolumab was the strongest source of variation in the ICERs. Probability sensitivity analysis showed that the probability for the PD-L1 test-based strategy being cost-effective increases with the increase of WTP thresholds.

**Conclusion:**

From the perspective of the Chinese healthcare system, using a PD-L1 test to guide second-line nivolumab treatment was not cost-effective. The National Healthcare Security Administration negotiation on the price reduction of nivolumab was found to be the most effective action to improve its cost-effectiveness in China.

## Introduction

Lung cancer remains a major public health problem and the most commonly diagnosed cancer in China that contributes to 27% of all cancer-related deaths (1). Non-small cell lung cancer (NSCLC) accounts for approximately 85% of lung cancer cases, and most of them are advanced cases (2). In the pre-immunotherapy era, the prognosis of advanced NSCLC was generally poor, and the 5-year survival rate was less than 5.5% (3). The popularity of immunotherapy for treating advanced NSCLC has significantly prolonged the overall survival of patients with advanced NSCLC (4, 5). Since 2018, immune checkpoint inhibitors (ICIs) have been successively approved by the Chinese government as the standard treatment for advanced NSCLC, and the new therapeutic classes have presented favorable treatment efficacy and safety (6).

>Nivolumab as the first programmed death 1 (PD-1) ICI, was officially authorized by the Chinese State Food and Drug Administration (SFDA) as a second-line therapy for NSCLC in June 2018 (7). The crucial evidence underpinning the approval of nivolumab was yielded from the CheckMate 078 Phase III clinical trials, in which nivolumab was found that significantly improved the overall survival (OS) in NSCLC patients compared with docetaxel (the median OS: 12.0 vs 9.6 months) (8). In addition, this study found that the nivolumab therapy was more effective in treating advanced NSCLC with a programmed death ligand 1 (PD-L1) tumor proportion score (TPS) ≥1% (the median OS: 12.3) (8). Although the National Comprehensive Cancer Network (NCCN) guidelines of the United States recommend routine testing for PD-L1 expression in patients with diagnosed advanced NSCLC, and PD-L1 expression has been demonstrated as a reliable biomarker to predict benefits from immunotherapy (9, 10), there is lack of such recommendation in relevant Chinese treatment guidelines (11).

In 2015, China reported 733,300 new lung cancer cases, of which nearly 60% were advanced NSCLCs (1). From a Chinese healthcare system perspective, our previous cost-effectiveness analysis revealed that second-line nivolumab was unlikely to be cost-effective compared with docetaxel in patients with advanced NSCLC, despite the subgroup analysis showing the improved cost-effectiveness of nivolumab in the patients with PD-L1TPS ≥1% (12). Although this finding did not concur with the cost-effectiveness analyses conducted in other countries showing a favorable cost-effectiveness of nivolumab versus docetaxel in previously treated advanced NSCLC patients regardless of PD-L1 expression (13, 14), different perspectives, trial source used for analysis, and approach to modeling used between these studies have to be highlighted that may explain the inconsistency. Considering that nivolumab is recommended as the preferred second-line treatment for advanced NSCLC patients without *ALK* or *EGFR* mutations regardless of their PD-L1 expression in China, evidence regarding the impact of PD-L1 test results on the comparative cost-effectiveness of second-line nivolumab versus docetaxel from a Chinese health system perspective is urgently needed to inform Chinese healthcare policy making.

## Materials and Methods

### Model Structure

This economic evaluation used aggregate data from the CheckMate-078 trial and was therefore exempted from institutional research ethics board approval. The model design followed the guidelines for pharmacoeconomic evaluation in China (15).

**Figure 1:**
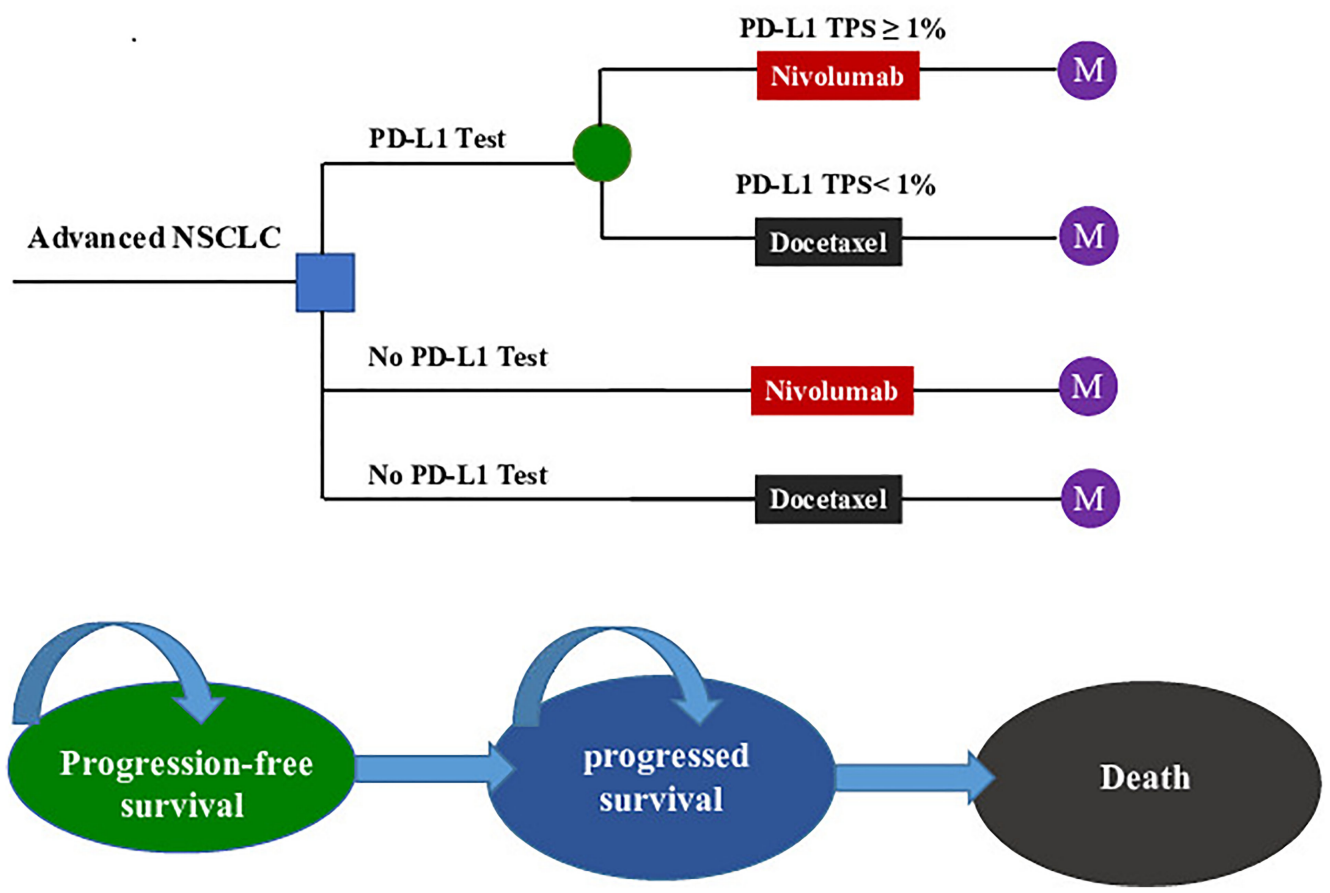
The Markov state transition model. NSCLC, non-small cell lung cancer; PD-L1, programmed death ligand 1; TPS, tumor proportion score; M, Markov node.

We established a Markov model consisting of three health states: progression-free survival state (PFS state), progressed survival state (PS state), and death to simulate the treatment and survival process for a cohort of Chinese NSCLC patients (**Figure 1**). The cost and effectiveness associated with the second-line treatments were estimated according to the transfer probability between different health states, and the medical expenses and health outcomes assigned to each health state. Our economic evaluation was conducted from the perspective of the Chinese healthcare system.

The target population was confirmed pre-treated advanced NSCLC patients who were negative for the *EGFR* mutation or *ALK* mutation. All patients started in the PFS state and could move to another health state according to transition probabilities. For two strategies without a PD-L1 test, nivolumab (A) and docetaxel (B) were randomly assigned to patients regardless of their PD-L1 expression. For the PD-L1 test-based strategy, patients were treated according to their PD-L1 status (C): patients with a PD-L1TPS ≥1% were assigned to receive nivolumab (C-Niv), and those who had a TPS <1% were assigned to receive docetaxel (C-Doc). We assumed that 55% of patients had a PD-L1TPS ≥1% (8). Second-line treatment regimens and dosages in the model followed those detailed in the CheckMate078 trial (**Supplementary Table 1**) (8). The primary analysis was preformed to compare (1) the PD-L1 test-based strategy (C) with docetaxel (A); (2) the PD-L1 test-based strategy (C) with nivolumab (B); and (3) the nivolumab (B) and docetaxel (A).

Medication schemes for nivolumab and docetaxel were adjusted to fit a 3-week model cycle. A lifetime horizon was used to project cancer treatment-related costs and health outcomes for this analysis. In this model, patients in PFS state were assumed to receive second-line nivolumab or docetaxel until disease progression, or discontinuation owing to toxicity. Subsequent therapy included chemotherapy, targeted therapy and immunotherapy was assigned to 42% of the patients in the treatment groups whose disease progressed. Other patients were recommended for the best supportive care (BSC) according to current clinical guidelines in China (16).

The principal output of our model was the incremental cost-effectiveness ratios (ICERs) between treatment strategies under comparison, which were calculated as the incremental costs per quality-adjusted life year (QALY) gained. A discount rate of 5% per year was recommended by the Guidelines for Pharmacoeconomic Evaluation in China (15). All the costs were reported in 2019 Chinese yuan and US dollars. Since there is no recommended willing-to-pay (WTP) threshold in Chinese pharmacoeconomic guidelines, we used three times the gross domestic product (GDP) as the WTP threshold according to the recommendation from the World Health Organization (WHO). In light of the imbalance in economic development among different regions in China, we compared ICERs with two WTP thresholds: CNY 212,667 (USD 30,828)/QALY [3 × the per capita gross domestic product (GDP) value of China in 2019] for general regions and CNY 492,656 (USD 71,415)/QALY (3 × the per capita GDP value of Beijing city in 2019) for affluent regions (17). In our study, all the analyses were performed with TreeAge Pro 2018 software (https://www.treeage.com/).

### Clinical Inputs

For the two strategies without PD-L1 test, we digitized the PFS and OS curves from the CheckMate 078 trial (ClinicalTrials.gov: NCT02613507) to extract clinical efficacy data points (8, 18). To minimize the impact of statistical fluctuations on our results, we constructed the pseudo-individual patient data based on Hoyle et al.’s algorithm (19). Then, the PFS and OS projections were modeled by fitting pseudo-individual patient data with four commonly used parameter distributions, namely, exponential, Weibull, log-logistic, and log-normal distributions.

For the PD-L1 test-based strategy, we digitized the OS curves for subgroups of nivolumab-treated patients with a PD-L1 TPS ≥1% and docetaxel-treated patients with a PD-L1 TPS <1% from the CheckMate 078 trial to extract clinical efficacy data points, and then fitted and extrapolated data points with the four commonly used parameter distributions. However, the PFS curves by tumor PD-L1 expression have not yet been published along with the results of the CheckMate 078 trial. Therefore, we assumed that the PFS data of these two subgroups were similar to those of the whole trial population corresponding to nivolumab or docetaxel treatment.

**Figure 2:**
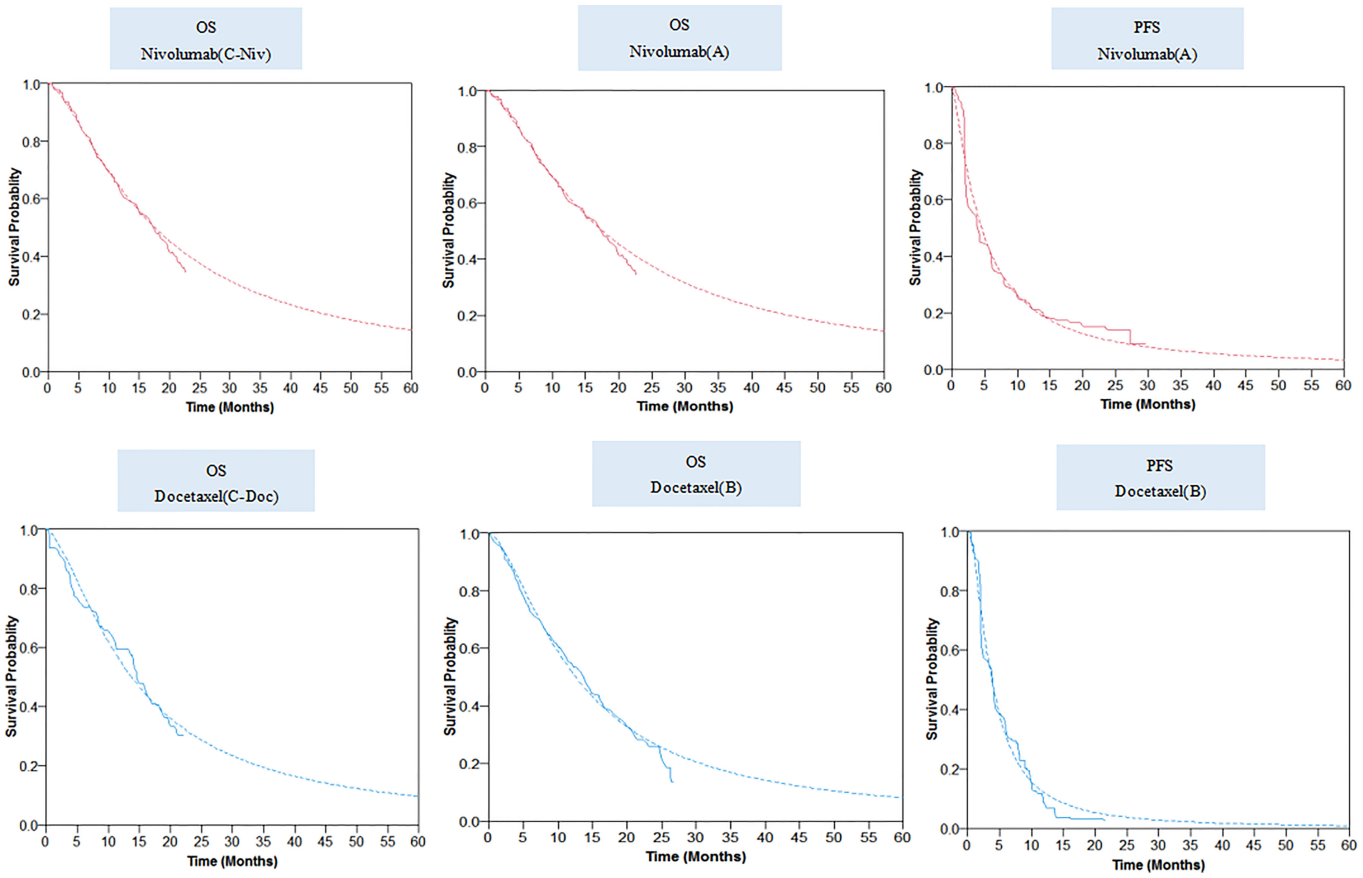
Parametric survival distributions fitted and extrapolated for OS and PFS data. The solid lines represented the observed data for each strategy; the dotted lines represented the fitted data for each strategy. OS, overall survival; PFS, progression-free survival.

The final log-logistic variables, theta (θ), and kappa (k) listed in **Table 1**, were estimated using R software (version 3.3.1, http://www.r-project.org). In this study, log-logistic distribution was chosen based on the result of goodness of fit test using the Akaike’s information criterion (AIC) and Bayesian information criterion (BIC) (**Supplementary Tables 2**, **3**). For the validation purposes, the predicted OS and PFS curves were compared with the investigated Kaplan–Meier (KM) curves (**Figure 2**).

**Table 1:** Model parameters: baseline values, ranges, and distributions for sensitivity analysis.

Parameter	Value	Range	Distribution	Ref
**Survival**				
**The PD-L1 test-based strategy (C)**				
Log-logistic OS survival of nivolumab (C-Niv)	Theta = 0.01532; kappa = 1.45712	–	–	(18)
Log-logistic OS survival of docetaxel (C-Doc)	Theta = 0.01816; kappa = 1.52881	–	–	(18)
Log-logistic PFS survival of nivolumab (C-Niv)	Theta = 0.01502; kappa = 1.49269	–	–	(8)
Log-logistic PFS survival of docetaxel (C-Doc)	Theta = 0.01925; kappa = 1.55954	–	–	(8)
**No PD-L1 test strategy**				
Log-logistic PFS survival of nivolumab (B)	Theta = 0.1402; kappa = 1.3017	–	–	(8)
Log-logistic PFS survival of docetaxel (A)	Theta = 0.1001; kappa = 1.7305	–	–	(8)
Log-logistic OS survival of nivolumab (B)	Theta = 0.01502; kappa = 1.49269	–	–	(8)
Log-logistic OS survival of docetaxel (A)	Theta = 0.01925; kappa = 1.55954	–	–	(8)
**Costs (**CNY)				
Nivolumab (4.5 mg/kg per cycle)	413.9	124.2–413.9	Fixed in PSA	Local charge
Docetaxel (75 mg/m^2^ per cycle)^a^	38.6	31.0–46.2	Fixed in PSA	Local charge
PD-L1 test	322.2	258.0–386.3	Lognormal	Local charge
Routine follow-up per cycle^b^	383.6	287.7–478.8	Lognormal	(20)
Subsequent therapy in PS state per cycle^c^	5,892.0	4,873.8–6,846.1	Lognormal	(21)
BSC per cycle^d^	2,328.2	1,862.6–2,793.9	Lognormal	(20)
Death-associated costs^e^	18,127.9	15,810.0–20,465.1	Lognormal	(21)
Neutropenia per event	3,183.7	2,865.6–3,502.4	Lognormal	(22)
Anemia per event	3,667.9	3,300.9–4,034.9	Lognormal	(22)
Fatigue per event	796.1	716.1–875.4	Lognormal	(22)
Rash per event	37.9	30.4–45.5	Lognormal	(23)
**Utilities**				
PFS state	0.768	0.614–0.922	Beta	(24)
PS state	0.703	0.562–0.844	Beta	(24)
Disutility for neutropenia	0.200	0.160–0.240	Beta	(25)
Disutility for fatigue	0.070	0.060–0.080	Beta	(25)
Disutility for rash	0.100	0.080–0.120	Beta	(25)
**Risk for treatment-related AEs**				
Neutropenia in nivolumab arm	0.003	0.002–0.004	Beta	(8)
Neutropenia in docetaxel arm	0.147	0.118–0.177	Beta	(8)
Anemia in nivolumab arm	0.003	0.002–0.004	Beta	(8)
Anemia in docetaxel arm	0.019	0.015–0.023	Beta	(8)
Fatigue in nivolumab arm	0.008	0.007–0.011	Beta	(8)
Fatigue in docetaxel arm	0.032	0.025–0.038	Beta	(8)
Rash in nivolumab arm	0.008	0.007–0.011	Beta	(8)
Rash in docetaxel arm	–	–	–	(8)
**Other**				
The proportion of PD-L1 TPS ≥1% (%)	0.55	0.44–0.66	Beta	(8)
Discount rate (%)	5	0–8	Fixed in PSA	(15)
Patient weight (kg)	65	52–78	Normal	(26)
body surface area (m^2^)	1.72	1.38–2.07	Normal	(26)

PD-L1, programmed death ligand 1; OS, overall survival; PFS, progression-free survival; PS, progressed survival; BSC, best supportive care; AEs, adverse effects; TPS, tumor proportion score.

a Docetaxel has been included in the category B list of the Chinese basic medical insurance drug list, the drug expenses incurred by treating advanced non-small-cell lung cancer patients with docetaxel need only be paid at 5% by the patients themselves, and the remaining 95% is paid by medical insurance.

b The cost of routine follow-up included the cost of outpatient physician visit, laboratory tests and examinations.

According to CheckMate 078 trial, subsequent therapy referred to the treatment after disease progression, including chemotherapy, targeted therapy and immunotherapy.

BSC referred to the intervention of clinical symptoms caused by cancer, the treatment of complications of anti-tumor treatment, and the rehabilitation treatment after the whole treatment.

Death-associated costs referred to the cost of palliative end-of-life.

The time-dependent transition probabilities of death were calculated according to the following formula:


$$tp_{die}=1-\frac{1+\exp(\theta_{OS}){\left(t-1\right)}^{k_{OS}}}{1+\exp(\theta_{OS})t^{k_{OS}}}(k>0),$$

The transition probabilities of PFS were calculated from the following formula:


$$tp_{pfs}=\frac{1+\exp(\theta_{pfs}){\left(t-1\right)}^{k_{pfs}}}{1+\exp(\theta_{pfs})t^{k_{pfs}}}/(1-tp_{die})$$

where *t* represents the current number of Markov model cycles (27).

### Cost Estimates

Costs associated with cancer treatment in the analysis only covered direct medical costs, namely, drug acquisition, PD-L1 test, treating major adverse events (AEs), routine follow-up, subsequent therapy, BSC, and death-associated costs (12, 28). The cost of nivolumab was obtained from the Chinese health industry big data service platform (https://db.yaozh.com/). The cost of docetaxel was calculated based on the local bid-winning price and the payment ratio of the Chinese basic medical insurance (see **Table 1** for details). In calculating dosage amounts, a mean body weight of 65 kg (range, 52–78 kg) and a body surface area of 1.72 m^2^ (range, 1.38–2.07 m^2^) were assumed in the model (26).

As per our previous study, costs for managing AEs associated with rash, fatigue, anemia, and neutropenia were considered in this economic analysis (12). Costs estimates for these AEs were derived from published studies (22, 23), and the risks were obtained from the CheckMate 078 trial (**Supplementary Table 4**). The costs of the PD-L1 test, as well as other costs related to cancer treatment were collected from the National Development and Reform Commission of China (29), local hospitals or published studies (20, 21). The cost inputs used in the model are detailed in **Table 1**.

### Utility Estimates

PFS and PS health state utilities were obtained from a published study that measured health utilities in Chinese NSCLC patients (24). The decrease in utility caused by treatment-related grade III/IV toxicities was considered in our model (25). Therefore, the utility value for PFS state in the economic evaluation was weighted by the risk of AEs reported in the CheckMate 078 trial, and the corresponding utility decreases. The utility values used in the model are listed in **Table 1**.

### Statistical Analysis

One-way deterministic sensitivity analyses (DSA) were conducted to determine the influence of uncertainties in individual input variables on our results. In general, model variables were tested within 95% confidence intervals quoted from the published literature or assumed to vary within ±20% of the base-case value (**Table 1**). Probabilistic sensitivity analyses (PSA) were performed by running 1,000 iterations to generate 1,000 estimates of costs and QALYs for each treatment strategy to test the robustness of our findings. For each iteration, model inputs varied simultaneously and were randomly sampled from appropriate statistical distributions (**Table 1**). The PSA results were presented by a cost-effectiveness acceptability curve (CEAC).

To explore the impact of economic and health policies with Chinese characteristics on our results, we conducted the following two scenario analyses. First, China has a large population and is a rapidly developed developing country, thus the imbalance in economic development among different province-level administrative units is an objective fact. The China Statistical Yearbook 2019 showed that the per capita GDP in the Chinese mainland varied widely from CNY 33,058 (USD 4,792) in Gansu Province to CNY 164,220 (USD 23,805) in Beijing city (17). Against such economic background, we explored the probability that the PD-L1 test-based strategy (C) is cost-effective when compared with alternative treatment strategies under different WTPs (3 × per capita GDP value of each province-level administrative unit). Second, to alleviate the economic burden on cancer patients, since 2017, the price of many cancer drugs has been reduced by 30–70% through the National Healthcare Security Administration (NHSA) negotiations over cancer drugs in China. Therefore, we paid more attention to the impact of the NHSA negotiations on our results. Scenario analyses were performed based on the 30 to 70% reduction in nivolumab price.

## Results

### Base-Case Analysis

In the PD-L1 test base case, the model projected a mean cost of CNY334,301 (USD 48,460) and a mean survival of 1.22 QALYs per patient for the PD-L1 test-based strategy (C), and the ICERs for the PD-L1 test-based strategy (C) vs docetaxel (A) and vs nivolumab (B), were estimated to be CNY 731,089 (USD 105,978) per QALY and CNY 2165,577 (USD 313,920) per QALY, respectively. The higher total direct medical costs associated with nivolumab were mainly attributed to the higher drug acquisition costs, which were significantly impacted by the improved PFS.

In the no PD-L1 test base case, the model projected a mean cost of CNY 459,833 (USD 66,657) and a mean survival of 1.27 QALYs per patient for nivolumab (B), while a mean cost of CNY 139,701 (USD 20,251) and the mean survival of 0.95 per patient for docetaxel (A), yielded an ICER of CNY 987,618 (USD 143,016)/QALY for nivolumab (B) vs docetaxel (A). The predicted mean costs and effectiveness related to each strategy are listed in **Table 2** for comparison.

**Table 2:** Summary base case results.

Model outputs	No PD-L1 test strategy		The PD-L1 test-based strategy			Incremental		
	Docetaxel (A)	Nivolumab (B)	Overall^a^ (C)	Docetaxel (C-Doc)	Nivolumab (C-Niv)	B vs A	C vs A	C vs B
**LYs**	1.33	1.75	1.69	1.46	1.87	0.42	0.36	−0.06
PFS state	0.42	0.68	0.57	0.42	0.69	0.26	0.15	−0.11
PS state	0.91	1.07	1.13	1.05	1.19	0.16	0.22	0.06
**QALYs**	0.95	1.27	1.22	1.04	1.36	0.32	0.27	−0.06
PFS state	0.31	0.52	0.43	0.31	0.52	0.21	0.12	−0.09
PS state	0.64	0.75	0.79	0.74	0.83	0.11	0.15	0.04
**Cost (CNY)**	139,699	459,836	334,302	158,262	478,335	320,137	194,607	−125,532
PD-L1 test cost	0	0	324	324	324	0	324	324
Drug acquisition cost	442	297,794	165,143	442	299,898	297,353	164,702	−132,644
Routine follow-up cost	3,365	3,932	3,235	2,345	3,960	1,587	890	−697
AEs management cost	566	28	269	566	28	−538	−297	241
Subsequent therapy cost^b^	91,460	107,348	112,528	104,754	118,882	15,715	20,889	5,174
BSC cost	36,210	42,419	44,468	41,398	46,979	6,209	8,258	2,042
Death-associated cost	8,499	8,313	8,340	8,444	8,258	−186	−159	28
**ICER (CNY)**								
Cost per LY						762,229	547,410	1,946,253
Cost per QALY						987,618	731,089	2,165,577

LY, life-year; QALY, quality-adjusted life-year; ICER, incremental cost-effectiveness ratio; PFS, progression-free survival; PS, progressed survival; PD-L1, programmed death ligand 1; AEs, adverse effects; BSC, best supportive care.

a The total mean costs and QALYs of overall patients in the PD-L1 test-based strategy were calculated by multiplying the proportion of patients with a PD-L1 TPS ≥1% (reported in the CheckMate 078 trial) by the mean cost and QALYs of nivolumab-treated patients, plus the proportion of patients with a PD-L1 TPS <1% (reported in the CheckMate 078 trial) multiplied by the mean cost and QALY of docetaxel-treated patients.

b Subsequent therapy costs in PS state were estimated based on the proportion of patients received subsequent after disease progressed reported in the CheckMate 078 trial.

In our WTP threshold scenario analysis, the estimated ICERs between the PD-L1 test-based strategy (C) and docetaxel (A) were higher than the WTPs defined based on the different per capita GDP in Chinese mainland. In our price reduction scenario analysis, we found that reducing the price of nivolumab decreased the total medical costs for nivolumab-treated patients, therefore, to a great extent, significantly lowered the ICERs between nivolumab-treated arm and docetaxel-treated arm. **Supplementary Tables 5**, **6** show the results of price reduction and WTP scenario analyses.

### Sensitivity Analyses

#### Deterministic Sensitivity Analysis

**Figure 3:**
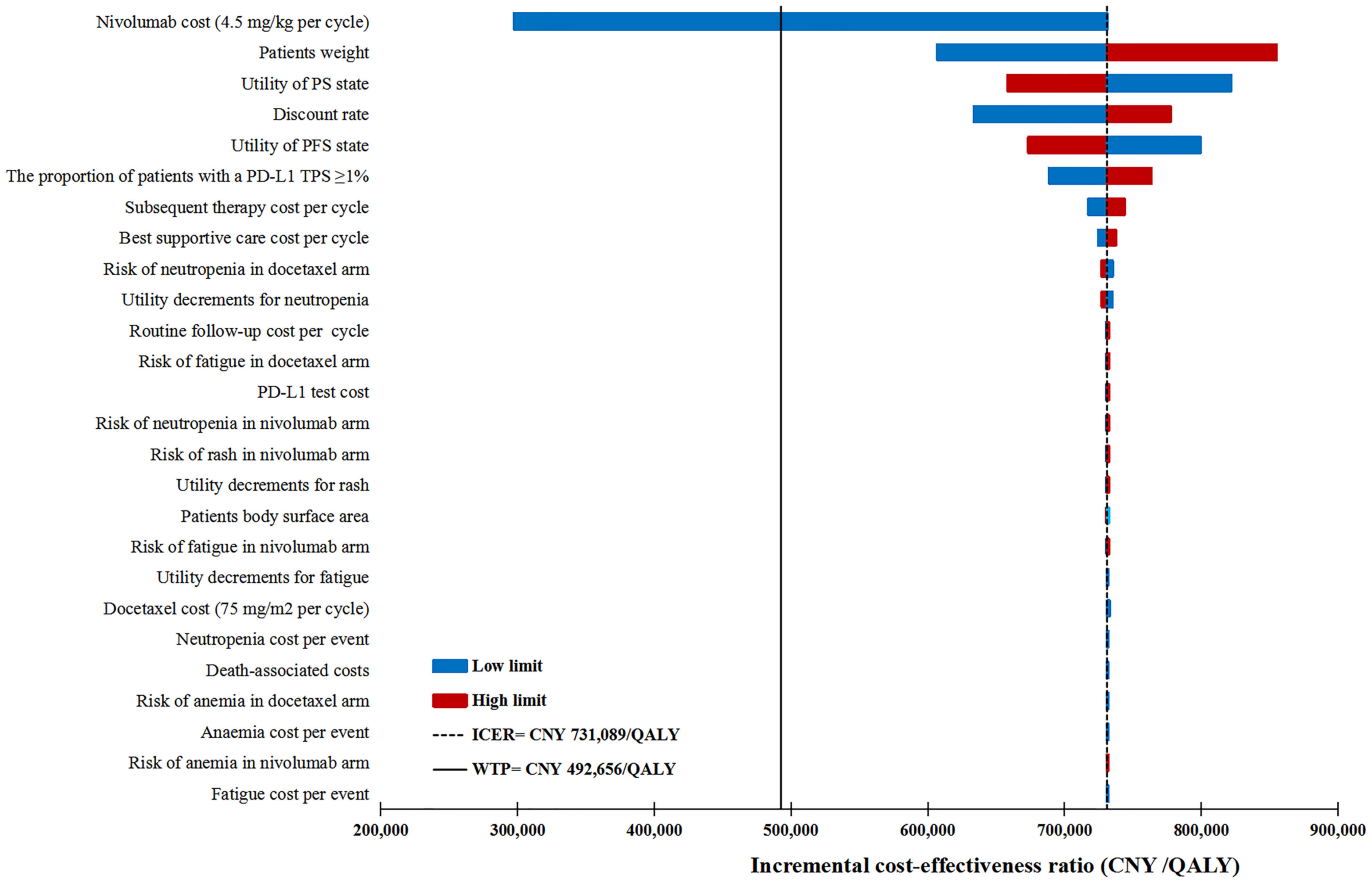
The result of one-way deterministic sensitivity analysis for the PD-L1 test-based strategy (C) versus docetaxel (A). ICER, incremental cost-effectiveness ratio; WTP, willingness-to-pay; QALY, quality-adjusted life-year; PFS, progression-free survival; PS, progressed survival; PD-L1, programmed death ligand 1; TPS, tumor proportion score.

The DSA results were visualized by tornado diagrams. The price of nivolumab was a main driver for the variation in ICERs. The ICERs decreased to a greater extent with the lower limit of the nivolumab price. In the PD-L1 test base case, the ICER between the PD-L1 test-based strategy (C) and docetaxel (A) dropped below the WTPs for affluent regions, when the reduction in the price of nivolumab exceeded 39% (**Figure 3**). In the no PD-L1 test base case, the ICER between nivolumab (B) and docetaxel (A) dropped below the WTPs for affluent regions, when the reduction in the price of nivolumab exceeded 54% (**Supplementary Figure 1**).

The patient weight, utility for PFS state, discount rate, proportion of patients with a PD-L1 TPS ≥1%, and the utility for the PS state also had considerable influences on the ICERs. Other variables, namely, the risk of AEs, costs other than drug acquisition cost, and decreased utility related to grade III/IV AEs had minimal influence on the ICERs. The results indicated that the lower or upper limits of any tested variable failed to result in the ICERs for the PD-L1 test-based strategy (C) vs docetaxel (A) to be lower than the WTP for general regions. However, the lower limits of the cost of nivolumab (4.5 mg/kg per cycle) produced an ICER below the WTP for affluent regions.

#### Probabilistic Sensitivity Analysis

**Figure 4:**
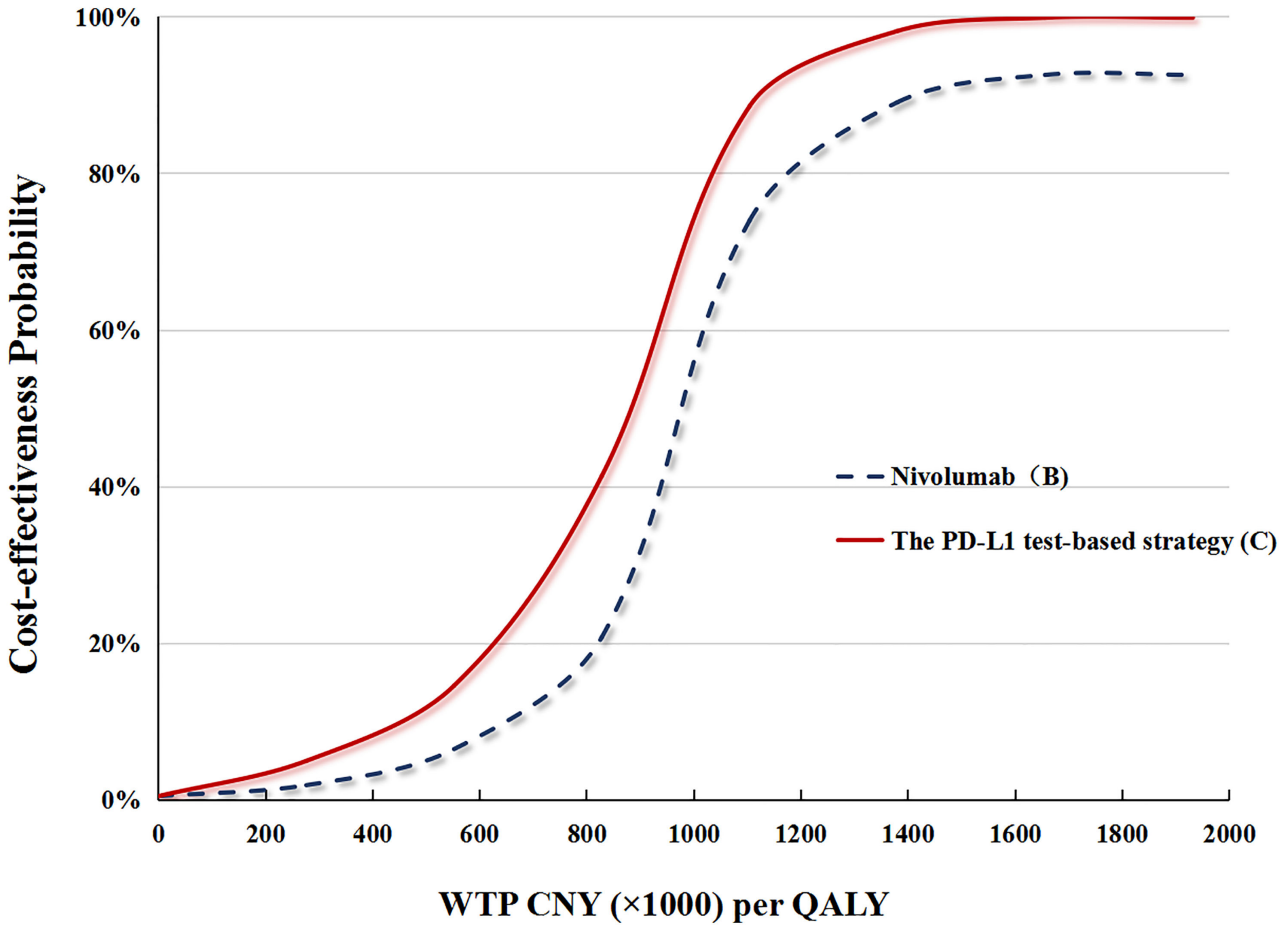
**(A)** The cost-effectiveness probability achieved by the PD-L1 test-based strategy (C) and nivolumab (B) compared to docetaxel (A) at different WTP thresholds. QALY, quality adjusted life-year; WTP, willingness-to-pay; PD-L1, programmed death ligand.

In performing the PSA for the PD-L1 test base case, compared with docetaxel (A), the cost-effective probabilities of PD-L1 test-based strategy (C) were 16 and 4% when the WTP was CNY 212,667 (USD 30,828)/QALY and CNY 492,656 (USD 71,415)/QALY, respectively (**Figure 4**).

In performing the PSA for the no PD-L1 test base case, compared with docetaxel (A), cost-effective probabilities of nivolumab (B) were nearly 14% when WTP was CNY 492,656 (USD 71,415)/QALY, and zero when WTP was CNY 212,667 (USD 30,828)/QALY (**Figure 4**).

**Figure 5:**
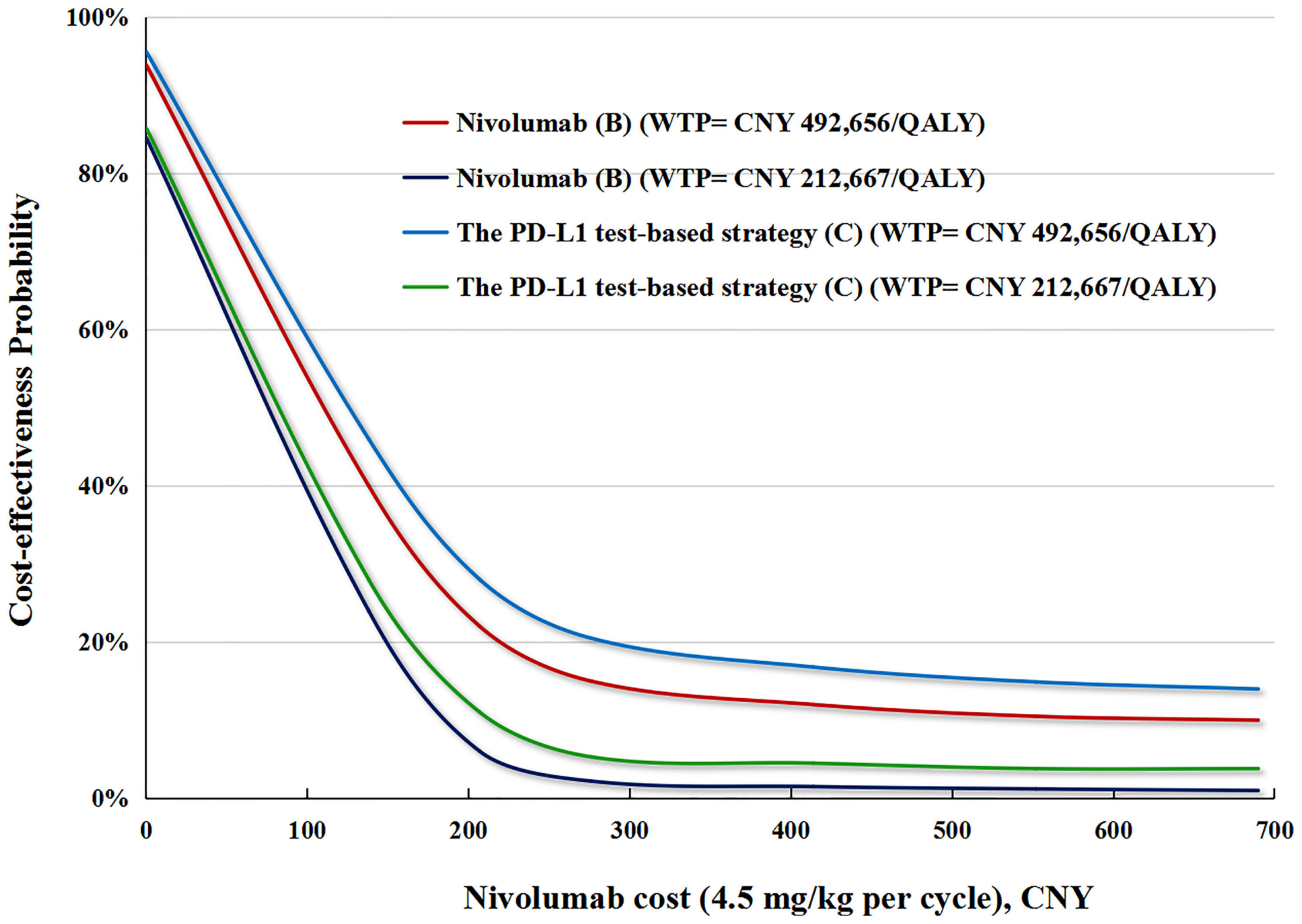
The cost-effectiveness probability achieved by the PD-L1 test-based strategy (C) and nivolumab (B) compared to docetaxel (A) at different nivolumab prices. QALY, quality adjusted life-year; WTP, willingness-to-pay; PD-L1, programmed death ligand.

In the price reduction scenario, the possibility of the nivolumab strategy being cost-effective increased as the nivolumab price decreased. In the PD-L1test base case, a 50% reduction in the price of nivolumab increased the cost-effective probability of the PD-L1 test-based strategy (C) to up to 26 and 6%, respectively, at the WTPs of CNY492,656 (USD 71,415)/QALY and CNY 212,667 (USD 30,828)/QALY. In the no PD-L1test base case, a 50% reduction in the price of nivolumab increased the cost-effective probability of nivolumab (B) to up to 19 and 4% at the WTPs of CNY492,656 (USD 71,415)/QALY and CNY 212,667 (USD 30,828)/QALY, respectively (**Figure 5**).

## Discussion

Our study was the first economic evaluation investigating the costs and health outcomes of using the PD-L1 test to guide second-line nivolumab treatment for Chinese advanced NSCLC patients with no *EGFR* mutations or *ALK* translocations. Our results demonstrated that compared with docetaxel (A), the PD-L1 test-based strategy (C) extended survival in PFS and PS states by 0.12 QALYs and 0.15 QALYs, respectively (see detail in **Table 2**). Using two WTPs in this study, we found that the incremental costs of the PD-L1 test-based strategy (C) [CNY334,301 (USD 48,460) vs CNY139,702 (USD 20,251)] were not commensurate with the modest survival benefits it can provide, when compared with the docetaxel (A). As a result, the ICERs were not in favor of the PD-L1 test-based strategy (C).

The expert consensus on immunosuppressive therapy for NSCLC in China (2019) recommends nivolumab monotherapy as the preferred second-line treatment for advanced NSCLC with no *EGFR* mutations or *ALK* translocations, regardless of PD-L1 expression (30). Despite this, the expression of PD-L1 was found to be related to the efficacy of nivolumab (8). Hence, in this study, we aimed to advance the discussion around whether employing the PD-L1 test to guide second-line nivolumab therapy is cost-effective. The PD-L1 test-based strategy (C) was associated with greater survival benefits than nivolumab (B), mainly because that the nivolumab is more effective in advanced NSCLC with high levels of PD-L1 expression (8). Although we concluded that the PD-L1 test-based strategy (C) was not cost-effective compared with docetaxel (A), it produced a lower ICER than nivolumab (B). These results suggested that selecting patients for second-line nivolumab based on the PD-L1 test result improved its cost-effectiveness. The current study did not evaluate the cost-effectiveness of nivolumab in patients with higher PD-L1 expression, due to the lack of relevant clinical data.

Comprehensive sensitivity analyses were performed to assess the robustness of our model. Reducing the price of nivolumab was found to be the most realistic action to push the PD-L1 test-based strategy (C) toward cost-effectiveness. In recent years, great efforts have been paid to reduce the price of anticancer drugs through the negotiation with pharmaceutical companies held by the NHSA in China, and as a result, the prices of many anticancer drugs have dropped by 30 to 70% (31). Therefore, negotiation over nivolumab might be an effective way to promote the cost-effectiveness of the PD-L1 test-based strategy (C) in China. In the long run, the NHSA negotiation, which enables patients to obtain better treatment at lower cost, will be the most attainable approach for optimizing medical resource allocation in China. Moreover, the WTP threshold scenario analysis showed that with the increase of the WTP threshold value, the PD-L1 test-based strategy (C) were more cost-effective in China, which were generally consistent with our previous study (12). To reflect China’s regional economic disequilibrium, two WTP thresholds were selected for general regions and affluent regions in the current analyses, respectively.

Pharmacoeconomic evaluation evidence regarding the PD-L1 test was rather limited. Only one study from the Swiss healthcare setting assessed the impact of the PD-L1 test on the cost-effectiveness of nivolumab (32). This analysis used the CheckMate-057 trial as the source for clinical inputs and reported an ICER of CHF 124,891/QALY for nivolumab in patients with a TPS ≥10%. Our results cannot be directly compared with it because the different clinical inputs sources and different study perspectives were used. However, they concluded that the cost-effectiveness of nivolumab was improved by selecting patients according to the consequences of the PD-L1 test. This finding was consistent with ours. Additionally, our previous analysis assessing the cost-effectiveness of CheckMate 078 comparators reported an ICER of CNY 643,678 (USD 93,307)/QALY for second-line nivolumab vs docetaxel, which is much lower than our current results (12). The inconsistency of the ICERs might result from the fact that our current study used the latest 2-year follow-up data from CheckMate 078 (16), which were not available in our previous study.

Our study has the following strengths. First, we synthesized the latest 2-year follow-up data of the CheckMate 078 trial through economic modeling to project the costs and health outcomes associated with second-line nivolumab and docetaxel, bolstering the reliability of these cost-effectiveness results. Second, our economic evaluation considered the cost-effectiveness of second-line nivolumab and docetaxel in different PD-L1 statuses that provided comprehensive and accurate economic profiles of the two therapies. We applied two WTP thresholds in the model, reflecting the cost-effectiveness of the PD-L1 test-based strategy (C) in both high-income and resource-constrained regions of China. By contrasting and discussing our analysis results, this paper presents proposals for the PD-L1 test-based strategy (C) to serve the patients most likely to benefit from it.

Our study has several limitations. *First*, KM survival curves obtained from the CheckMate 078 trial clinical trial were used to project survivals. Any biases in this trial, if existed, would have inevitably been reflected in our model. *Second*, a potential bias in our Markov model was that the local data on the prevalence of PD-L1 TPS ≥1% were not available due to the lack of relevant studies in China. *Third*, as previously mentioned, we only considered the costs of grade III/IV AEs affecting ≥10% of patients reported in the CheckMate 078 trial, which might lead to an uncertainty in the estimation of AE costs. However, DSA, performed by varying model inputs within a broad range, found that the ICERs were not quite sensitive to AE costs. *Fourth*, the current study did not consider other ICIs, such as pembrolizumab, which is a potential comparator for advanced NSCLC without *EGFR* and *ALK* mutations. One reason is the lack of head-to-head clinical trials. The second reason is that in China, nivolumab is limited to the second-line treatment for advanced NSCLC patients, while pembrolizumab is not. *Fifth*, there is an uncertainty in the long-term survival projection beyond the trial period, more mature data are needed to validate our model against longer-term survival data. *Finally*, generalizing our study findings to other countries/regions might be difficult.

In conclusion, for pretreated advanced NSCLC patients with no EGFR mutations or ALK translocations, using the PD-L1 test to guide second-line nivolumab treatment might not be considered cost-effective from the perspective of the Chinese healthcare system. Reducing the price of nivolumab was found to be the most realistic action to push nivolumab strategies toward cost-effectiveness.

## Data Availability Statement

The original contributions presented in the study are included in the article/**Supplementary Material**. Further inquiries can be directed to the corresponding authors.

## Author Contributions

XZ and QL had full access to all the data in the study and take responsibility for the integrity of the data and the accuracy of the data analysis. Concept and design: QL, XZ, and CT. Acquisition, analysis, or interpretation of data: All authors. Drafting of the manuscript: QL, XZ, and CT. Critical revision of the manuscript for important intellectual content: All authors. Statistical analysis: QL. Obtained funding: QL. Supervision: XZ and CT. All authors contributed to the article and approved the submitted version.

## Funding

This work was supported by the Hunan Provincial Natural Science Foundation (grant number 2019JJ50864); and the Scientific research project of Hunan Health Commission in 2019 (grant number B2019156).

## Conflict of Interest

The authors declare that the research was conducted in the absence of any commercial or financial relationships that could be construed as a potential conflict of interest.

## Publisher’s Note

All claims expressed in this article are solely those of the authors and do not necessarily represent those of their affiliated organizations, or those of the publisher, the editors and the reviewers. Any product that may be evaluated in this article, or claim that may be made by its manufacturer, is not guaranteed or endorsed by the publisher.

## Supplementary Material

Supplementary Figure 1 The result of one-way deterministic sensitivity analysis. The tornado diagram revealed the influence of uncertainty in individual model input variables on the ICERs between nivolumab **(B)** versus docetaxel **(A)**. The black dotted line represented the ICERs estimated from our base case analysis, and the black solid line represented the WTP of CNY492,656 (71,415 USD)/QALY for affluent regions in China. ICER, incremental cost-effectiveness ratio; WTP, willingness-to-pay; QALY, quality-adjusted life-year; PFS, progression-free survival; PS, progressed survival.

Supplementary Table 1 Second-line treatment regimens and dosage in the Model.

Supplementary Table 2 Parametric survival distributions fitted for OS data.

Supplementary Table 3 Parametric survival distributions fitted for PFS data.

Supplementary Table 4 Incidence of treatment-Related grade III/IV Adverse Events considered in the model.

Supplementary Table 5 The results of WTP threshold scenario analysis. The WTP threshold values used in the scenario analyses were set on the basis of the per capita gross domestic product (GDP) of different province-level administrative unit in Mainland Chinese.

Supplementary Table 6 The results of the price reduction scenario analysis. The price reduction scenario analyses were conducted based on the price of nivolumab discounted ranging from 30% and 70%.
