# Supplementary figures and images for: PD-L1 Test-Based Strategy With Nivolumab as the Second-Line Treatment in Advanced NSCLC： A Cost-Effectiveness Analysis in China
Source: Front Oncol. 2021 Dec 13;11:745493. doi: 10.3389/fonc.2021.745493 (PMC8710478; doi:10.3389/fonc.2021.745493)

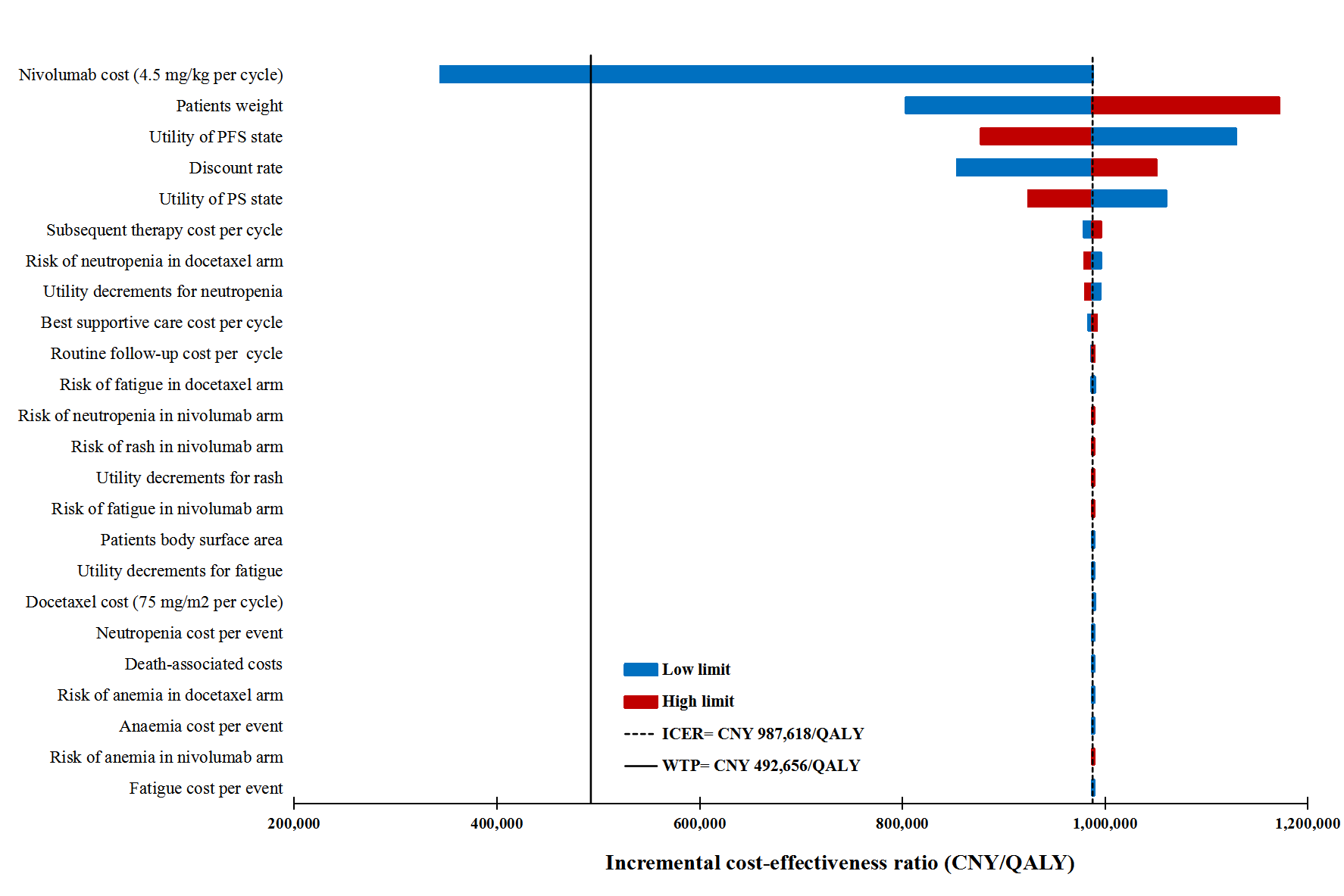

Supplement: Supplementary Figure 1 — The result of one-way deterministic sensitivity analysis. The tornado diagram revealed the influence of uncertainty in individual model input variables on the ICERs between nivolumab (B) versus docetaxel (A). The black dotted line represented the ICERs estimated from our base case analysis, and the black solid line represented the WTP of CNY492,656 (71,415 USD)/QALY for affluent regions in China. ICER, incremental cost-effectiveness ratio; WTP, willingness-to-pay; QALY, quality-adjusted life-year; PFS, progression-free survival; PS, progressed survival. [file Image_1.tif]
