# Supplementary material for: PD-L1 Test-Based Strategy With Nivolumab as the Second-Line Treatment in Advanced NSCLC： A Cost-Effectiveness Analysis in China
Source: Front Oncol. 2021 Dec 13;11:745493. doi: 10.3389/fonc.2021.745493 (PMC8710478; doi:10.3389/fonc.2021.745493)
Supplement: Supplementary Table 1 — Second-line treatment regimens and dosage in the Model. [file Table_1.doc]

Table 1. Second-line treatment regimens and dosage in the Model

| **Regimens** | **Dosage** | **Frequency** |
| --- | --- | --- |
| **No PD-L1 test base case** |  |  |
| Nivolumab for all patients | 3 mg/kg | Day 1 of every 2-week cycle |
| Docetaxel for all patients | 75 mg/m^2^ | Day 1 of every 3-week cycle |
| **PD-L1 test base case** |  |  |
| Nivolumab Tumor PD-L1 expression≥ 1% | 3 mg/kg | Day 1 of every 2-week cycle |
| Docetaxel Tumor PD-L1 expression< 1% | 75 mg/m^2^ | Day 1 of every 3-week cycle |

*PD-L1, programmed death ligand 1.*
