# Supplementary material for: PD-L1 Test-Based Strategy With Nivolumab as the Second-Line Treatment in Advanced NSCLC： A Cost-Effectiveness Analysis in China
Source: Front Oncol. 2021 Dec 13;11:745493. doi: 10.3389/fonc.2021.745493 (PMC8710478; doi:10.3389/fonc.2021.745493)
Supplement: Supplementary Table 2 — Parametric survival distributions fitted for OS data. [file Table_2.doc]

**Table 2. Parametric survival distributions fitted for OS data.**

| **Parametric Model** | **No PD-L1 test base case** | | | | **PD-L1 test base case** | | | |
| --- | --- | --- | --- | --- | --- | --- | --- | --- |
|  | **Nivolumab** | | **Docetaxel** | | **Nivolumab** | | **Docetaxel** | |
|  | **AIC** | **BIC** | **AIC** | **BIC** | **AIC** | **BIC** | **AIC** | **BIC** |
| Exponential | -571.0 | -565.3 | -401.4 | -396.3 | -578.9 | -573.1 | -469.7 | -464.3 |
| Weibull | -754.3 | -745.8 | -510.7 | -503.0 | -599.6 | -590.9 | -471.8 | -463.7 |
| Lognormal | -717.2 | -708.7 | -406.5 | -398.8 | -893.3 | -884.7 | -435.7 | -427.6 |
| Loglogistic | -754.5 | -746.0 | -418.0 | -410.3 | -873.3 | -864.6 | -450.9 | -442.8 |

*OS, overall survival; PD-L1, programmed death ligand 1; AIC, Akaike’s information criterion; BIC, Bayesian information criterion.*
