# Supplementary material for: PD-L1 Test-Based Strategy With Nivolumab as the Second-Line Treatment in Advanced NSCLC： A Cost-Effectiveness Analysis in China
Source: Front Oncol. 2021 Dec 13;11:745493. doi: 10.3389/fonc.2021.745493 (PMC8710478; doi:10.3389/fonc.2021.745493)
Supplement: Supplementary Table 3 — Parametric survival distributions fitted for PFS data. [file Table_3.doc]

**Table 3. Parametric survival distributions fitted for PFS data.**

| **Parametric Model** | **Nivolumab** | | **Docetaxel** | |
| --- | --- | --- | --- | --- |
|  | **AIC** | **BIC** | **AIC** | **BIC** |
| Exponential | -374.9 | -368.7 | -531.2 | -525.0 |
| Weibull | -407.2 | -397.9 | -571.1 | -561.8 |
| Lognormal | -502.7 | -493.4 | -608.2 | -598.9 |
| Loglogistic | -507.4 | -498.1 | -582.2 | -572.9 |

*PFS, progression-free survival; AIC,Akaike’s information criterion; BIC, Bayesian information criterion.*
