# Supplementary material for: PD-L1 Test-Based Strategy With Nivolumab as the Second-Line Treatment in Advanced NSCLC： A Cost-Effectiveness Analysis in China
Source: Front Oncol. 2021 Dec 13;11:745493. doi: 10.3389/fonc.2021.745493 (PMC8710478; doi:10.3389/fonc.2021.745493)
Supplement: Supplementary Table 4 — Incidence of treatment-Related grade III/IV Adverse Events considered in the model. [file Table_4.doc]

**Table 4. Incidence of treatment-Related grade III/IV Adverse Events considered in the model.**

| **Patients, n (%)** | **Nivolumab (n=337)** | **Docetaxel (n =156)** |
| --- | --- | --- |
| Rash | 3 (1) | 0 |
| Fatigue | 3 (1) | 5 (3) |
| Anemia | 1 (<1) | 3 (2) |
| Neutropenia | 1 (<1) | 23 (15) |
