# Supplementary material for: PD-L1 Test-Based Strategy With Nivolumab as the Second-Line Treatment in Advanced NSCLC： A Cost-Effectiveness Analysis in China
Source: Front Oncol. 2021 Dec 13;11:745493. doi: 10.3389/fonc.2021.745493 (PMC8710478; doi:10.3389/fonc.2021.745493)
Supplement: Supplementary Table 5 — The results of WTP threshold scenario analysis. The WTP threshold values used in the scenario analyses were set on the basis of the per capita gross domestic product (GDP) of different province-level administrative unit in Mainland Chinese. [file Table_5.doc]

**Table 5. The results of WTP threshold scenario analysis**

| **Region** | | **Per-capita GDP (CNY)** | **WTP (3×Per-capita GDP, CNY)** | **ICERs compared with different WTPs** | | |
| --- | --- | --- | --- | --- | --- | --- |
|  |  |  |  | **Nivolumab (B)**  **vs Docetaxel(A)** | **The PD-L1 test-based strategy(C) vs Docetaxel(A)** | **The PD-L1 test-based strategy(C) vs Nivolumab (B)** |
| Mainland China | | 70,889 | 212,667 | higher | higher | **lower** |
| 1 | Beijing | 164,219 | 492,656 | higher | higher | **lower** |
| 2 | Shanghai | 157,424 | 472,271 | higher | higher | **lower** |
| 3 | Jiangshu | 123,752 | 371,257 | higher | higher | **lower** |
| 4 | Zhejiang | 108,686 | 326,058 | higher | higher | **lower** |
| 5 | Fujian | 107,575 | 322,726 | higher | higher | **lower** |
| 6 | Guangdong | 94,896 | 284,687 | higher | higher | **lower** |
| 7 | Tianjing | 90,432 | 271,297 | higher | higher | **lower** |
| 8 | Hubei | 77,449 | 232,348 | higher | higher | **lower** |
| 9 | Chongqing | 75,828 | 227,485 | higher | higher | **lower** |
| 10 | Shandong | 70,730 | 212,191 | higher | higher | higher |
| 11 | Neimenggu | 67,930 | 203,789 | higher | higher | higher |
| 12 | Shanxi | 66,743 | 200,229 | higher | higher | higher |
| 13 | Anhui | 58,692 | 176,077 | higher | higher | higher |
| 14 | Hunan | 57,623 | 172,870 | higher | higher | higher |
| 15 | Liaoning | 57,140 | 171,421 | higher | higher | higher |
| 16 | Hainan | 56,823 | 170,469 | higher | higher | higher |
| 17 | Henan | 56,775 | 170,324 | higher | higher | higher |
| 18 | Sichuan | 55,885 | 167,654 | higher | higher | higher |
| 19 | Xinjiang | 54,678 | 164,033 | higher | higher | higher |
| 20 | Ningxia | 54,477 | 163,432 | higher | higher | higher |
| 21 | Jiangxi | 53,270 | 159,811 | higher | higher | higher |
| 22 | Xizang | 49,379 | 148,138 | higher | higher | higher |
| 23 | Qinghai | 49,166 | 147,497 | higher | higher | higher |
| 24 | Yunnan | 48,089 | 144,268 | higher | higher | higher |
| 25 | Guizhou | 46,579 | 139,736 | higher | higher | higher |
| 26 | Hebei | 46,454 | 139,363 | higher | higher | higher |
| 27 | Shanxi | 45,792 | 137,377 | higher | higher | higher |
| 28 | Jilin | 43,364 | 130,092 | higher | higher | higher |
| 29 | Guangxi | 43,116 | 129,347 | higher | higher | higher |
| 30 | Heilongjiang | 36,079 | 108,237 | higher | higher | higher |
| 31 | Gansu | 33,058 | 99,173 | higher | higher | higher |

*GDP, the gross domestic product; WTP, willingness-to-pay; ICER, incremental cost-effectiveness ratio.*
