# Supplementary material for: PD-L1 Test-Based Strategy With Nivolumab as the Second-Line Treatment in Advanced NSCLC： A Cost-Effectiveness Analysis in China
Source: Front Oncol. 2021 Dec 13;11:745493. doi: 10.3389/fonc.2021.745493 (PMC8710478; doi:10.3389/fonc.2021.745493)
Supplement: Supplementary Table 6 — The results of the price reduction scenario analysis. The price reduction scenario analyses were conducted based on the price of nivolumab discounted ranging from 30% and 70%. [file Table_6.doc]

**Table 6. The results of the price reduction scenario analysis**

|  | **Mean cost (CNY)** | **Mean QALYs** | **Compared with** | **Incremental cost (CNY)** | **Incremental QALYs** | **ICER (CNY)** |
| --- | --- | --- | --- | --- | --- | --- |
| **30% reduction in nivolumab’ price** | | | | | | |
| Nivolumab (B) | 370,498 | 1.27 | Docetaxel(A) | 230,797 | 0.32 | 721,240 |
| The PD-L1 test-based strategy(C) | 284,818 | 1.22 | Docetaxel(A) | 145,117 | 0.27 | 537,472 |
|  |  |  | Nivolumab (B) | -85,679 | -0.06 | 1,427,990 |
| **40% reduction in nivolumab’ price** | | | | | | |
| Nivolumab (B) | 340,717 | 1.27 | Docetaxel(A) | 201,016 | 0.32 | 628,175 |
| The PD-L1 test-based strategy(C) | 268,324 | 1.22 | Docetaxel(A) | 128,623 | 0.27 | 476,382 |
|  |  |  | Nivolumab (B) | -72,393 | -0.06 | 1,206,548 |
| **50% reduction in nivolumab’ price** | | | | | | |
| Nivolumab (B) | 310,943 | 1.27 | Docetaxel(A) | 171,242 | 0.32 | 535,131 |
| The PD-L1 test-based strategy(C) | 251,830 | 1.22 | Docetaxel(A) | 112,129 | 0.27 | 415,292 |
|  |  |  | Nivolumab (B) | -59,113 | -0.06 | 985,221 |
| **60% reduction in nivolumab’ price** | | | | | | |
| Nivolumab (B) | 281,162 | 1.27 | Docetaxel(A) | 141,461 | 0.32 | 442,066 |
| The PD-L1 test-based strategy(C) | 235,335 | 1.22 | Docetaxel(A) | 95,634 | 0.27 | 354,202 |
|  |  |  | Nivolumab (B) | -45,827 | -0.06 | 763,779 |
| **70% reduction in nivolumab’ price** | | | | | | |
| Nivolumab (B) | 251,381 | 1.27 | Docetaxel(A) | 111,680 | 0.32 | 349,001 |
| The PD-L1 test-based strategy(C) | 218,841 | 1.22 | Docetaxel(A) | 79,140 | 0.27 | 293,112 |
|  |  |  | Nivolumab (B) | -32,540 | -0.06 | 542,337 |

*QALY, quality-adjusted life-year; ICER, incremental cost-effectiveness ratio.*
